# Machine learning and medical education

**DOI:** 10.1038/s41746-018-0061-1

**Published:** 2018-09-27

**Authors:** Vijaya B. Kolachalama, Priya S. Garg

**Affiliations:** 1 Section of Computational Biomedicine, Department of Medicine, Boston University School of Medicine, Boston, MA 02118 USA; 2 Whitaker Cardiovascular Institute, Boston University School of Medicine, Boston, MA 02118 USA; 3 Hariri Institute for Computing and Computational Science and Engineering, Boston University, Boston, MA 02215 USA; 4 Medical Education Office, Boston University School of Medicine, Boston, MA 02118 USA

**Keywords:** Translational research, Education

## Abstract

Artificial intelligence (AI) driven by machine learning (ML) algorithms is a branch in computer science that is rapidly gaining popularity within the healthcare sector. Recent regulatory approvals of AI-driven companion diagnostics and other products are glimmers of a future in which these tools could play a key role by defining the way medicine will be practiced. Educating the next generation of medical professionals with the right ML techniques will enable them to become part of this emerging data science revolution.

## Introduction

Artificial intelligence (AI) is poised to help deliver precision medicine and health.^1,2^ The clinical and biomedical research communities are increasingly embracing this modality to develop tools for diagnosis and prediction as well as to improve delivery and effectiveness of healthcare. New breakthroughs are being developed in an unprecedented fashion and the developed ones have obtained regulatory approval and found their way into routine medical practice.^3–5^ Yet, the medical school curriculum as well as the graduate medical education and other teaching programs within academic hospitals across the United States and around the world have not yet come to grips with educating students and trainees on this emerging technology. Several expert opinions have pointed to the benefits and limitations associated with the use of ML in medicine,^1,2,6–10^ but the aspect related to formally educating the younger generation of medical professionals has not been openly discussed.

### Rise of the machines

**Fig. 1:**
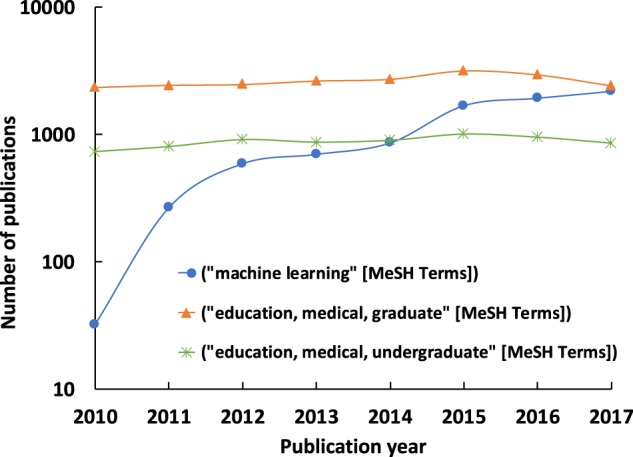
Published papers within this decade as listed on US National Library of Medicine (PubMed) using “machine learning”, “education, medical, graduate”, and “education, medical, undergraduate” as MeSH terms, respectively. The actual user queries were: (i) “machine learning”[MeSH Terms] and (“2010/01/01”[PDAT]: “2017/12/31”[PDAT]), (ii) “education, medical, graduate”[MeSH Terms] and (“2010/01/01”[PDAT]: “2017/12/31”[PDAT]), and (iii) “education, medical, undergraduate”[MeSH Terms] and (“2010/01/01”[PDAT]: “2017/12/31”[PDAT])

**Fig. 2:**
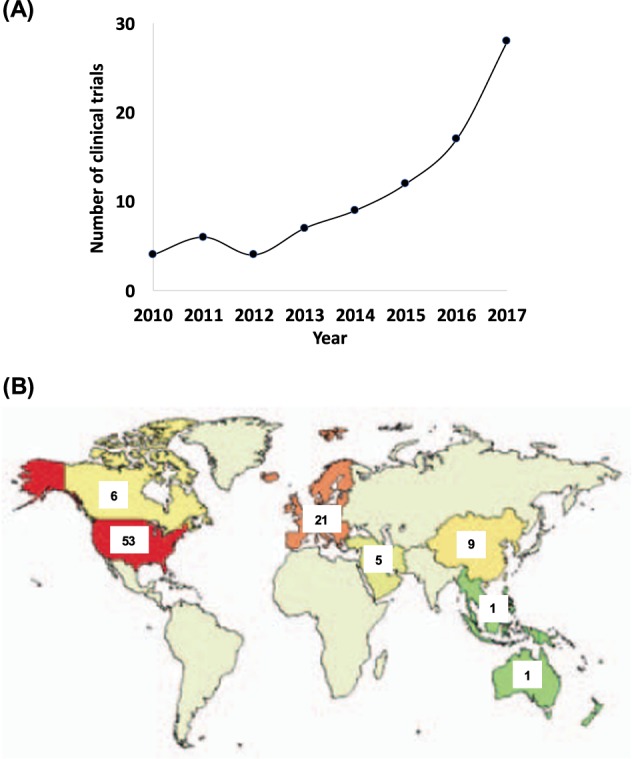
**a** Number of clinical trials registered on an annual basis on the US National Library of Medicine with “machine learning” as the search term. **b** Distribution of the number of registered clinical trials in several countries around the world till date with “machine learning” as the search term. Data for the two plots were generated by fixing the recruitment status to: “not yet recruiting”, “recruiting”, “enrolling by invitation”, “active, not recruiting”, and “completed”

The rising popularity of machine learning (ML) techniques for medical applications is evident from the increasing amount of research conducted on this topic, the number of products that are obtaining regulatory approvals as well as the entrepreneurial efforts in this space over the past few years. A PubMed search with “machine learning” as the MeSH term shows that the number of papers published in the area of ML is largely increasing since the beginning of this decade (Fig. 1). On the other hand, the number of publications related to undergraduate and graduate medical education have remained relatively unchanged since 2010. A combined search using the MeSH terms “machine learning” and “graduate medical education” between 2010 and 2017 resulted in 16 publications. Detailed review of these papers revealed that none of them were actually focused on ML education for medical professionals. Also, a similar search on the ClinialTrials.gov website with “machine learning” as the keyword showed an increase in the number of registered clinical trials on an annual basis since the beginning of this decade (Fig. 2a). Further inspection revealed that these clinical trials were registered by organizations from various countries around the world (Fig. 2b). Lastly, it is interesting to note that healthcare has arguably one of the highest number of venture capital (VC) backed AI startups, and this figure is gradually increasing. VC funding for healthcare AI companies was about $3.6 billion in the last 5 years.^11^ The above facts underscore the increasing appreciation of the value that ML can potentially bring to the medical community. If this trend continues, then we may very well see a large number of AI-driven products and technologies integrated within the healthcare ecosystem in the coming decades. The question then remains as to whether a medical professional is willing to embrace these tools as part of their repertoire and if so, how they can get educated in terms of knowing the “art” as well as the “science” of the ML algorithms driving these technologies.

### Lack of student access to ML content

Lack of direct access to appropriate ML education for clinicians and biomedical researchers is not surprising. Multiple factors contribute to the failure of ML to be embedded within undergraduate and graduate medical education training. Currently, there are no accreditation requirements related to AI. Additionally, medical schools are already struggling to maintain curricular hours in the current schema with ever growing biomedical knowledge and calls for new content areas. In the United States, assessment in undergraduate medical education, which drives much of learning, is largely focused on preparation for licensing exams and a recent competency focus on entrustable professional activities (EPA’s), neither of which include AI.^12,13^ To add to this dilemma, similar to the early experience with quality improvement and patient safety education, medical schools lack the faculty expertise required to teach this content which is largely taught in the computer science, mathematics and engineering faculties. Lack of mentorship and faculty role modeling poses a significant challenge as students move from the preclinical to clinical environment and try to develop understanding of how AI knowledge can be applied and used in the clinical setting. AI impacts patients and patient care. Therefore, ML and its applications should be taught within medical school and needs to be formalized to train the next generation of clinicians and biomedical scientists to face data-driven challenges that can directly impact patient care in the coming decades.

### Curricular recommendations

Any curriculum designed to address ML should aim for machine learning literacy rather than proficiency. ML-related content can be embedded within a larger curricular construct that is focused on competence in using information technology to improve patient care. Although the natural inclination for most schools would be to focus training on how to use technology like the electronic health record (EHR), curricular leaders should be cautioned. Information technology, including ML curricula in medical education could begin with a focus on population health and the impact it can have on disease prediction, risk stratification, and management. Students could initially be introduced to ML through courses focused on population health and evidence-based medicine in which ML becomes an additional tool for the clinician to provide care. As stated in the EPA’s, the entrustable student would be “able to identify and use several available databases, search engines, or other appropriate tools, resulting in a manageable volume of information, most of which is relevant to the clinical question”.^14^ Students in the preclinical phase could be introduced to studies and databases that have highlighted the impact of natural language processing, data science, and thus the impact of ML on healthcare systems.^15,16^ Simulated ML and EHR platforms should be taught simultaneously while students practice searching data and asking questions. Trainees should also learn the benefits, risks, and the ethical dilemmas that exist when using ML. As they transition to the clinical years, ML should become experiential and students should be exposed to already developed and tested diagnostic tools that could potentially be used in hospitals at the point of care. When students gain experience in using ML-based diagnostic tools, they would begin to recognize the conditions and future applications where AI could potentially benefit clinical decision making and management.

There is also an opportunity to develop healthcare leaders with expertize in big data and ML. Additional coursework should be developed through electives, as part of leadership or business tracks, and combined programs such as MD/MBA, MD/MPH, and MD/PhD. Students participating in these advanced experiences may spend more time understanding the role of computer programming and developing coding skills. This does not mean that students should have extensive experience in programming but they should be open to learning a programming language and writing some computer codes during the class and beyond. In today’s day and age, this task is not impossible because adoption of ML by several scientific communities has led to the development of free, user-friendly software and other educational content that is easily understandable to general audience and available over the internet (i.e., GitHub, Medium, Stack Overflow, etc.). Programming skills would allow students to consider alternative careers in addition to the practice of medicine which may help to more rapidly integrate ML into medicine.

### Suggestions to the ML course instructor

At most schools, the instructor will most likely be a data scientist. Therefore, the ML expert needs to limit the use of heavy jargon in the classroom, if possible. Mathematical and computational underpinnings that are seemingly overwhelming to the inexperienced trainee need to be explained in a simple fashion. The intent of instruction should be to not expose trainees with tedious definitions or equations but teach them the concepts and make them comfortable with the tools so that they can begin to tackle a new data challenge without getting bogged down by the terminology. Students can be a lot more attentive and focus on the concepts when they do not feel the need to quickly search the meaning of a technical term over the internet during the class. The instructor should also recommend practical guidelines to choosing the right tools. Lectures and tutorials should be filled with real-world clinical examples. This is where the instructor’s creativity would come to play. Relevant examples along with provision of datasets and healthcare challenges would facilitate students to gain the practical know-how needed to quickly and powerfully apply these techniques to new problems.

While this article is just meant to provide an outline and a potential curricular structure to embed ML content within a medical school, this list is by no means exhaustive. Also, things not covered here are the type of ML techniques and assessments that should be included throughout training. When students complete this introductory experience, they should be in a position to confidently ask a clinical question, analyze the AI tools that exist, and approach several types of biomedical datasets using various ML techniques.

## Conclusion

It is time for medical schools to consider including content focused on ML and its applications as part of their curriculum. Medical students, residents, and fellows should have knowledge of ML and data science during their training period. This will only become a reality when medical schools begin to create curricular time for ML with an acknowledgment of the changes to come in healthcare, and there is no better time to do it than now.

## Electronic supplementary material

Data set

## Data Availability

Datasets were derived from public resources (https://www.ncbi.nlm.nih.gov/pubmed and https://clinicaltrials.gov).

## Electronic supplementary material

**Supplementary information** accompanies the paper on the *npj Digital Medicine* website (10.1038/s41746-018-0061-1).

## References

[1] Obermeyer Z, Emanuel EJ (2016). Predicting the future - big data, machine learning, and clinical medicine. N. Engl. J. Med..

[2] Darcy AM, Louie AK, Roberts LW (2016). Machine learning and the profession of medicine. JAMA.

[3] Gulshan V (2016). Development and validation of a deep learning algorithm for detection of diabetic retinopathy in retinal fundus photographs. JAMA.

[4] Ting DSW (2017). Development and validation of a deep learning system for diabetic retinopathy and related eye diseases using retinal images from multiethnic populations with diabetes. JAMA.

[5] *FDA permits marketing of artificial intelligence-based device to detect certain diabetes-related eye problems*, https://www.fda.gov/NewsEvents/Newsroom/PressAnnouncements/ucm604357.htm (2018).

[6] Chen JH, Asch SM (2017). Machine learning and prediction in medicine - beyond the peak of inflated expectations. N. Engl. J. Med..

[7] AI diagnostics need attention. *Nature* **555**, 285 (2018) https://www.ncbi.nlm.nih.gov/pubmed/29542717. 10.1038/d41586-018-03067-x 29542717

[8] Obermeyer Z, Lee TH (2017). Lost in thought - the limits of the human mind and the future of medicine. N. Engl. J. Med..

[9] Beam, A. L. & Kohane, I. S. Big data and machine learning in health care. *JAMA*10.1001/jama.2017.18391 (2018). 10.1001/jama.2017.18391 29532063

[10] Cabitza F, Rasoini R, Gensini GF (2017). Unintended consequences of machine learning in medicine. JAMA.

[11] *AI In Healthcare Heatmap: From Diagnostics To Drug Discovery, Deals Heats Up*, https://www.cbinsights.com/research/artificial-intelligence-healthcare-heatmap-expert-intelligence/ (2018).

[12] Wartman SA, Combs CD (2018). Medical education must move from the information age to the age of artificial intelligence. Acad. Med..

[13] Lomis K (2017). Implementing an entrustable professional activities framework in undergraduate medical education: early lessons from the AAMC core entrustable professional activities for entering residency pilot. Acad. Med..

[14] Beam AL, Kohane IS (2016). Translating artificial intelligence into clinical care. JAMA.

[15] Wald HS, George P, Reis SP, Taylor JS (2014). Electronic health record training in undergraduate medical education: bridging theory to practice with curricula for empowering patient- and relationship-centered care in the computerized setting. Acad. Med..

[16] Bates DW, Gawande AA (2003). Improving safety with information technology. N. Engl. J. Med..

